# Subtle white matter intensity changes on fluid-attenuated inversion recovery imaging in patients with ischaemic stroke

**DOI:** 10.1093/braincomms/fcae089

**Published:** 2024-03-13

**Authors:** Pedro Cougo, Heber Colares, João Gabriel Farinhas, Mariana Hämmerle, Pedro Neves, Raquel Bezerra, Alex Balduino, Ona Wu, Octavio M Pontes-Neto

**Affiliations:** Instituto Americas, Neurology Division, Rio de Janeiro 22775-001, Brazil; Hospital Samaritano Barra, Department of Neurology, Rio de Janeiro 22775-001, Brazil; Hospital Samaritano Barra, Department of Radiology, Rio de Janeiro, 22775-001, Brazil; Instituto Americas, Neurology Division, Rio de Janeiro 22775-001, Brazil; Hospital Samaritano Barra, Department of Neurology, Rio de Janeiro 22775-001, Brazil; Hospital Samaritano Barra, Department of Neurology, Rio de Janeiro 22775-001, Brazil; Hospital Samaritano Barra, Department of Radiology, Rio de Janeiro, 22775-001, Brazil; Hospital Samaritano Barra, Department of Radiology, Rio de Janeiro, 22775-001, Brazil; Instituto Americas, Neurology Division, Rio de Janeiro 22775-001, Brazil; Athinoula A. Martinos Centre for Biomedical Imaging, Massachusetts General Hospital, Charlestown, MA 02129, USA; Faculdade de Medicina de Ribeirão Preto, Universidade de São Paulo, Ribeirão Preto 14040-900, Brazil

**Keywords:** cerebral small-vessel disease, leukoaraiosis, free water, ischaemic stroke

## Abstract

Leukoaraiosis is a neuroimaging marker of small-vessel disease that is characterized by high signal intensity on fluid-attenuated inversion recovery MRI. There is increasing evidence from pathology and neuroimaging suggesting that the structural abnormalities that characterize leukoaraiosis are actually present within regions of normal-appearing white matter, and that the underlying pathophysiology of white matter damage related to small-vessel disease involves blood–brain barrier damage. In this study, we aim to verify whether leukoaraiosis is associated with elevated signal intensity on fluid-attenuated inversion recovery imaging, a marker of brain tissue free-water accumulation, in normal-appearing white matter. We performed a cross-sectional study of adult patients admitted to our hospital with a diagnosis of acute ischaemic stroke or transient ischaemic attack. Leukoaraiosis was segmented using a semi-automated method involving manual outlining and signal thresholding. White matter regions were segmented based on the probabilistic tissue maps from the International Consortium for Brain Mapping 152 atlas. Also, normal-appearing white matter was further segmented based on voxel distance from leukoaraiosis borders, resulting in five normal-appearing white matter strata at increasing voxel distances from leukoaraiosis. The relationship between mean normalized fluid-attenuated inversion recovery signal intensity on normal-appearing white matter and leukoaraiosis volume was studied in a multivariable statistical analysis using linear mixed modelling, having normal-appearing white matter strata as a clustering variable. One hundred consecutive patients meeting inclusion and exclusion criteria were selected for analysis (53% female, mean age 68 years). Mean normalized fluid-attenuated inversion recovery signal intensity on normal-appearing white matter was higher in the vicinity of leukoaraiosis and progressively lower at increasing distances from leukoaraiosis. In a multivariable analysis, the mean normalized fluid-attenuated inversion recovery signal intensity on normal-appearing white matter was positively associated with leukoaraiosis volume and age (*B* = 0.025 for each leukoaraiosis quartile increase; 95% confidence interval 0.019–0.030). This association was found similarly across normal-appearing white matter strata. Voxel maps of the mean normalized fluid-attenuated inversion recovery signal intensity on normal-appearing white matter showed an increase in signal intensity that was not adjacent to leukoaraiosis regions. Our results show that normal-appearing white matter exhibits subtle signal intensity changes on fluid-attenuated inversion recovery imaging that are related to leukoaraiosis burden. These results suggest that diffuse free-water accumulation is likely related to the aetiopathogenic processes underlying the development of white matter damage related to small-vessel disease.

## Teaser

Cougo *et al*. report that mean fluid-attenuated inversion recovery (FLAIR) signal intensity within normal-appearing white matter is increased in patients with larger volumes of leukoaraiosis in those with a history of ischaemic stroke. This increase in FLAIR signal intensity is non-adjacent to leukoaraiosis borders and points to diffuse free-water leakage related to small-vessel disease.

## Graphical Abstract

**Graphical Abstract:**
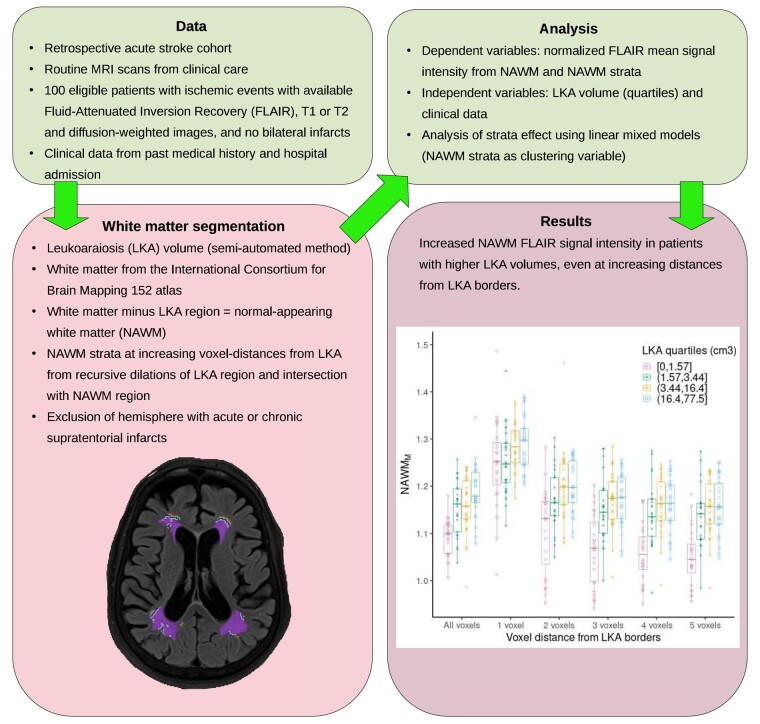

## Introduction

Leukoaraiosis is a neuroimaging marker of white matter damage related to small-vessel disease that is highly prevalent and associated with stroke incidence, cognitive decline and increased mortality.^1,2^ Furthermore, it has been associated with well-established vascular risk factors, such as age and hypertension, as well as clinical and tissue outcomes after acute stroke, including penumbral tissue loss, long-term functional outcome and stroke recurrence.^3-10^

Leukoaraiosis is defined as conspicuous regions of brain white matter that are hyperintense on T_2_-weighted MRI and that typically follow either a periventricular or a subcortical patchy distribution.^11^ These hyperintense regions are especially conspicuous on the fluid-attenuated inversion recovery (FLAIR) MRI sequence. However, a large body of literature has shown that leukoaraiosis-related changes in both pathology and neuroimaging can also be found in normal-appearing white matter (NAWM) and that white matter damage related to small-vessel disease encompasses neuroimaging changes beyond the conspicuous regions of white matter hyperintensity. Many of these studies have used diffusion tensor imaging (DTI) techniques, showing that abnormal diffusion values in NAWM were more pronounced in patients with more severe leukoaraiosis.^12,13^ Moreover, these studies have shown that DTI abnormalities in NAWM were diffuse and not restricted to the vicinity of leukoaraiosis regions.

While it seems clear that white matter damage is not restricted to leukoaraiosis regions, the biological processes underlying the development of both leukoaraiosis and NAWM damage have remained open to debate. From a pathophysiological perspective, the DTI changes related to white matter damage could represent either tissue compartment alterations, with the disorganization of the orientation of axonal fibres, or extravascular water accumulation. It has been argued that the underlying mechanism of vascular white matter disease would involve endothelial dysfunction and blood–brain barrier disruption. In studies evaluating blood–brain barrier disruption with dynamic gadolinium-enhanced and perfusion MRI, patients with small-vessel disease presented with extravascular leakage in NAWM that was related to the burden of leukoaraiosis.^14,15^ We hypothesize that if diffuse DTI abnormalities and blood–brain barrier disruption imaging markers on NAWM share the same aetiopathogenic process, then leukoaraiosis would be associated with diffuse extravascular fluid leakage. Given that FLAIR hyperintensity is a putative marker of tissue fluid accumulation, we aim to study (i) whether the severity of leukoaraiosis would be related to an increase in signal intensity on NAWM and (ii) whether this relationship would vary according to the proximity to the leukoaraiosis region.

## Materials and methods

### Subjects

This study was approved by our Institutional Review Board with a waiver of informed consent. We retrospectively selected eligible patients from a prospective, hospital-based, clinical stroke registry. The registry screens all adult patients admitted to our institution with a clinical diagnosis of stroke of any type. For this study, we reviewed patients from January 2020 to July 2022, who were admitted with a diagnosis of ischaemic stroke or transient ischaemic attack and who had an MRI performed at any time during the hospital admission, including FLAIR and diffusion-weighted imaging and either T_2_- or T_1_-weighted imaging. Patients with bilateral infarcts (either acute or chronic) or motion artefacts were excluded.

### Imaging acquisition and analysis

**Figure 1:**
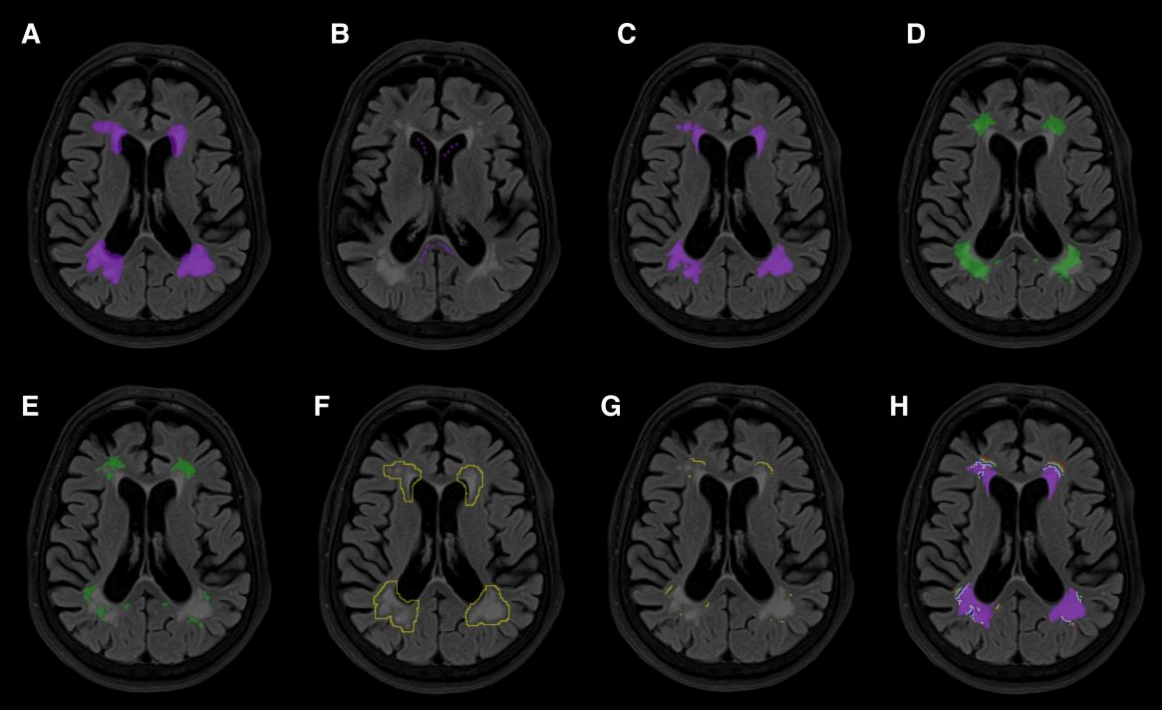
**A segmentation of leukoaraiosis and normal-appearing white matter.** (**A**) Leukoaraiosis (LKA) segmentation was obtained by manually outlining a broad region of interest (ROI) including all potential regions of LKA. (**B**) Secondly, 8–10 regions of interest of 1-mm diameter were placed in the *splenium* and anterior horn of the lateral ventricles. The final LKA mask (**C**) was obtained by thresholding the manually outlined mask to 6 SD above the mean signal intensity obtained from the splenium regions. Subtracting the final LKA volume from atlas-based white matter masks (**D**) resulted in the final normal-appearing white matter mask (**E**). Voxels with values below the maximum value obtained from the lateral ventricles were excluded from the final masks of LKA and NAWM. The third one-voxel dilation of LKA (**F**), which intersected with the final NAWM mask (**E**), generated the NAWM region at a three-voxel distance from LKA borders (**G**). (**H**) LKA and all NAWM strata according to voxel distance from LKA are depicted.

MRI was performed on a 3-T Siemens, Skyra scanner. Echo time, repetition time and inversion time for FLAIR images were 72, 9000 and 2500 ms. The slice thickness was 3.5 mm, the field-of-view was 210 mm × 240 mm and the matrix was 280 × 320. An imaging analysis was performed using the Medical Image NetCDF toolkit, version 1.0.08 for Linux.^16^ DICOM files were converted to MINC-2 format and anonymized. All images were corrected for intensity non-uniformity using the N3 method with parameter optimization for 3-T machines.^17,18^ MRI post-processing is illustrated in Fig. 1. T_2_- or T_1_-weighted and FLAIR images were co-registered to the International Consortium for Brain Mapping 152 atlas using linear and non-linear techniques.^19,20^ Given that these were images from standard clinical care, with a suboptimal resolution, we opted for a conservative approach for segmentation and thus prioritized specificity for white matter definition. For white matter segmentation, we used probabilistic tissue maps from the International Consortium for Brain Mapping 152 atlas with a threshold of 95% probability and included only supratentorial white matter.^21^ In order to avoid a misclassification of juxtacortical white matter, we restricted the white matter mask by applying consecutive operations of two erosions and one dilation,^22,23^ yielding a final mask that included mostly central white matter. White matter masks for each patient were obtained by an inverse co-registration of atlas masks to the original individual images. Leukoaraiosis masks were obtained from FLAIR images combining manual outlining and signal thresholding. The leukoaraiosis signal threshold was defined as 6 SD above the mean of splenium signal intensity, obtained from 8 to 10 regions of interest of 1-mm diameter. This threshold was derived by a study where FLAIR signal thresholding using a similar method and a 6-SD cut-off showed good intra-rater repeatability.^24^ However, in that study, the threshold values were related to slice selection of normal white matter, increasing from the brainstem to the vertex, and we therefore chose to use the same anatomical landmark for normal white matter reference selection (the splenium).

NAWM masks were then obtained by subtracting the leukoaraiosis mask from the white matter mask. For both leukoaraiosis and NAWM segmentation, voxels below the maximum signal intensity in CSF were excluded from the final masks, with this threshold obtained by sampling six to eight regions of interest of 1-mm diameter within the anterior horn of the lateral ventricles. This step was performed to exclude any signal from CSF from ventricular or cortical sulci regions originating from minor co-registration errors. Given our hypothesis that elevated FLAIR signal intensity would parallel the diffuse distribution of DTI changes described in other studies, we also obtained NAWM strata progressively distant from the leukoaraiosis borders. For this purpose, we performed five recursive, 2D, 8-kernel dilations of the leukoaraiosis mask. We then obtained the difference of each one from the predecessor and finally intersected the resulting mask with the NAWM mask (e.g. the NAWM mask at a three-voxel distance from leukoaraiosis was the result of the difference between the third leukoaraiosis dilation and the second leukoaraiosis dilation, intersected with the NAWM mask). This analysis of NAWM strata was also performed because we assumed that results from voxels outlining leukoaraiosis could possibly be contaminated by the choice of signal intensity cut-off for leukoaraiosis definition, and results from voxels distant from leukoaraiosis would be necessary for any reliable evaluation of the primary hypothesis. NAWM signal intensity was normalized to splenium mean signal intensity obtained from the above-mentioned splenium regions of interest. Finally, the mean normalized FLAIR signal intensity of the NAWM region (NAWM_M_) within each NAWM stratum was obtained for each patient for statistical analysis, as well as the total leukoaraiosis volume (LKA_V_). For both leukoaraiosis and NAWM segmentation, the infratentorial region was excluded. For patients with visible unilateral acute or chronic infarcts involving white matter, only the contralateral hemisphere was analysed for both NAWM and leukoaraiosis segmentation. In such cases, the total LKA_V_ measured in the unaffected hemisphere was doubled for statistical analysis. This conservative approach to exclude the hemisphere with visible infarcts, as opposed to masking out the infarct areas, was chosen because of the potential confluence between regions of FLAIR signal hyperintensity from both territorial and lacunar infarcts and regions of leukoaraiosis.

### Statistical analysis

For descriptive statistics, data are reported as mean (±standard deviation), median (interquartile range) or frequency. Since most patients did not have sagittal imaging, LKA_V_ could not be normalized to intracranial volume, and therefore, we used LKA_V_ quartiles as an ordinal variable in the analysis. The primary goal of the analysis was to evaluate the relationship between LKA_V_ and NAWM_M_. Underlying this analysis, two hypotheses were made: first, that baseline NAWM_M_ might vary according to the proximity to the leukoaraiosis, and second, also one of the specific aims of the study, that the relationship between NAWM_M_ and LKA_V_ might also vary according to the distance from the leukoaraiosis region. The first hypothesis was tested by comparing NAWM_M_ between NAWM strata with ANOVA. In order to test our second hypothesis, we performed a regression analysis by building linear mixed models, using NAWM_M_ as the dependent variable, LKA_V_ as the independent variable and NAWM strata as the clustering variable. In the first model, NAWM strata were entered only for random intercepts. In the second model, NAWM strata were entered for both random intercepts and random slopes. For model comparison, we compared Akaike information criteria and performed the likelihood test ratio of models built using the maximum likelihood method. The final reported models were built using the restricted maximum likelihood method. Considering the literature on leukoaraiosis risk factors, we included the age and history of hypertension in all models and any other variables related to NAWM_M_ in our sample. For that purpose, we performed a univariate analysis of the relationship between NAWM_M_ and all clinical variables using a two-sample *t*-test or Wilcoxon test for categorical variables and the Pearson’s or Spearman’s correlation coefficient for quantitative variables, as appropriate. Given the small number of patients with a history of anticoagulant use and heart failure, these variables were not included in the analysis. Statistical significance for all tests was determined by using a *P-*value of <0.05 on two-tailed tests. All statistical analyses were performed using R.

## Results

### Patients

Over the 36-month period, there were 317 cases of ischaemic stroke or transient ischaemic attack admitted to the hospital. The reasons for exclusion were absence of MRI (145), any combination of acute and/or chronic bilateral infarcts (43), absence of both T_2_- and T_1_-weighted imaging (24) and acquisition motion artefact (5), yielding a final sample of 100 patients (Table 1). LKA_V_ was positively associated with age (*ρ* = 0.43; *P* < 0.01), history of diabetes (*P* = 0.03) and dementia (*P* < 0.01).

**Table 1:** Study population

	Total (*n* = 100)
Age	68 ± 16
Female sex	53
Hypertension	68
Diabetes	32
Body mass index	27 ± 5
Obesity	19
Atrial fibrillation	7
Ischaemic heart disease	13
Prior statin use	27
Prior antiplatelet use	29
Infarct location	
Right hemisphere	24
Left hemisphere	31
Posterior fossa	11
No acute infarction	34
Leukaraiosis volume, cm^3^	3 (2–16)

Data are represented as mean ± standard deviation, frequency or median (interquartile range).

### NAWM_M_ analysis

**Figure 2:**
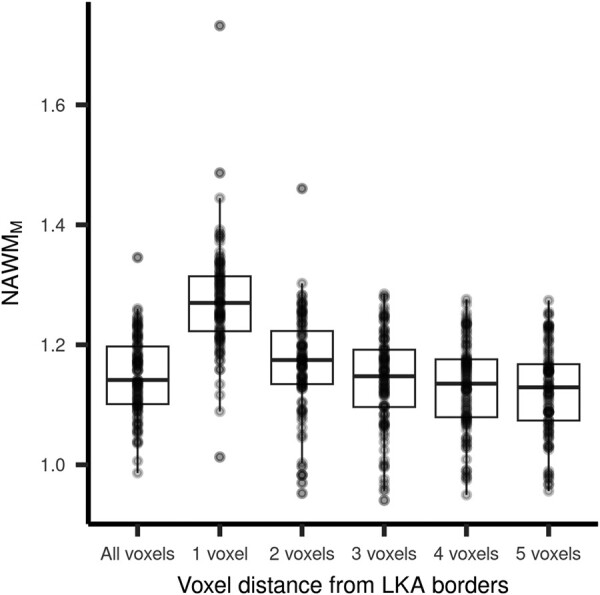
**NAWM_M_ at each NAWM stratum according to voxel distance from leukoaraiosis borders.** ANOVA: *F* = 52; *P* < 0.01.

NAWM_M_ was higher in the vicinity of leukoaraiosis borders and lower at stratum farther from leukoaraiosis (ANOVA: *F* = 52, *P* = 0.01; Fig. 2). In the univariate analysis, NAWM_M_ was associated with age (*r* = 0.26; *P* = 0.01) and LKA_V_ (*ρ* = 0.50; *P* < 0.01; Table 2). In the regression analysis using linear mixed modelling with random intercepts, the fixed effects of LKA_V_ and age were positively related to NAWM_M_. When entering random intercepts and random slopes in the model, the results for the fixed effects were very similar. The model with random intercepts [Akaike information criteria = (−1208)] and the model with random intercepts and random slopes [Akaike information criteria = (−1207)] performed similarly, and there was no significant difference in the likelihood test ratio (*P* = 0.14). The random effects estimates of LKA_V_ on NAWM_M_ for each NAWM stratum were all positive, numerically similar and close to the estimate from fixed effects, except for the first dilation, which showed a smaller estimate (Table 3).

**Table 2:** Group differences in NAWM_M_, univariate analysis

	NAWM_M_		
	No	Yes	*P*
Female sex	1.160 ± 0.067	1.140 ± 0.061	0.3
Hypertension	1.140 ± 0.067	1.150 ± 0.059	0.3
Diabetes	1.160 ± 0.069	1.140 ± 0.062	0.4
Atrial fibrillation	1.170 ± 0.065	1.17 ± 0.057	0.5
Ischaemic heart disease	1.150 ± 0.066	1.160 ± 0.055	0.6
Obesity	1.150 ± 0.068	1.150 ± 0.054	0.9
Dementia	1.150 ± 0.065	1.160 ± 0.045	0.4
Prior stroke	1.140 ± 0.061	1.170 ± 0.077	0.3
Prior statin use	1.150 ± 0.065	1.140 ± 0.064	0.6
Prior antiplatelet use	1.150 ± 0.066	1.160 ± 0.060	0.4

**Table 3:** A regression analysis using linear mixed modelling of NAWM_M_ using NAWM strata as the clustering variable

Linear mixed model with random intercepts			
	Estimate	Standard error	95% confidence interval
Fixed effects			
Intercept	1.026	0.031	0.096–1.090
Leukoaraiosis quartile increase	0.025	0.003	0.019–0.030
Age (each 10-year increase)	0.011	0.001	0.006–0.016
Hypertension	0.006	0.007	−0.008 to 0.020
Linear mixed model with random intercepts and random slopes			
	Estimate	Standard error	95% confidence interval
Fixed effects			
Intercept	1.026	0.037	0.947–1.104
Leukoaraiosis quartile increase	0.025	0.004	0.016–0.033
Age (each 10-year increase)	0.011	0.002	0.006–0.016
Hypertension	0.006	0.007	−0.008 to 0.020
Random effects	Intercept	Estimate	
Leukoaraiosis quartile increase, NAWM first voxel dilation	1.156	0.014	
Leukoaraiosis quartile increase, NAWM second voxel dilation	1.029	0.024	
Leukoaraiosis quartile increase, NAWM third voxel dilation	0.992	0.027	
Leukoaraiosis quartile increase, NAWM fourth voxel dilation	0.981	0.028	
Leukoaraiosis quartile increase, NAWM fifth voxel dilation	0.972	0.029	

**Figure 3:**
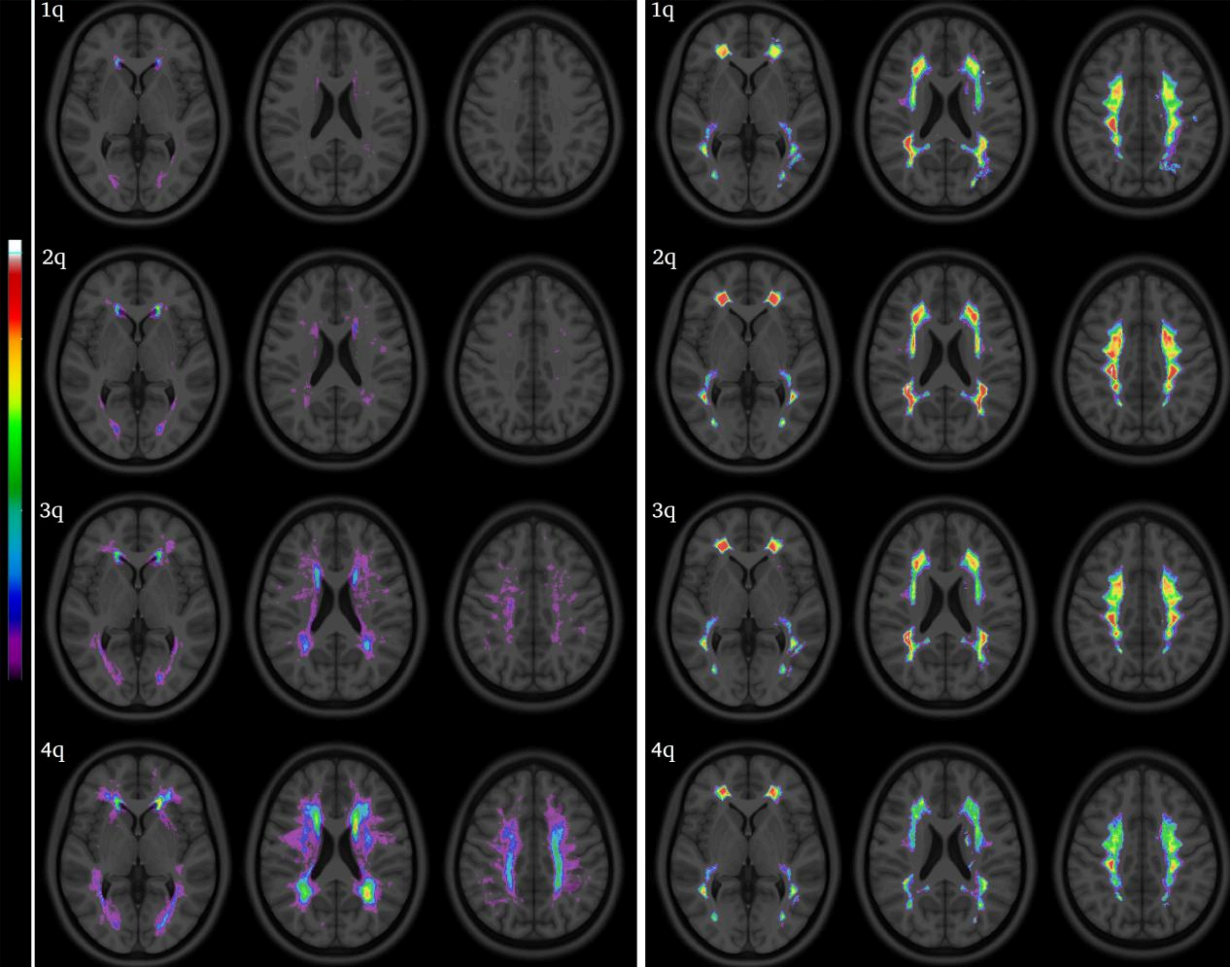
**Leukoaraiosis and mean normalized FLAIR signal intensity on NAWM in the Montreal Neurological Institute space.** The rows represent quartiles of leukoaraiosis volumes. The left panel represents leukoaraiosis frequency maps (spectral scale: 0–25). The right panel represents NAWM_M_ (spectral scale: 0.2–1.2).

**Figure 4:**
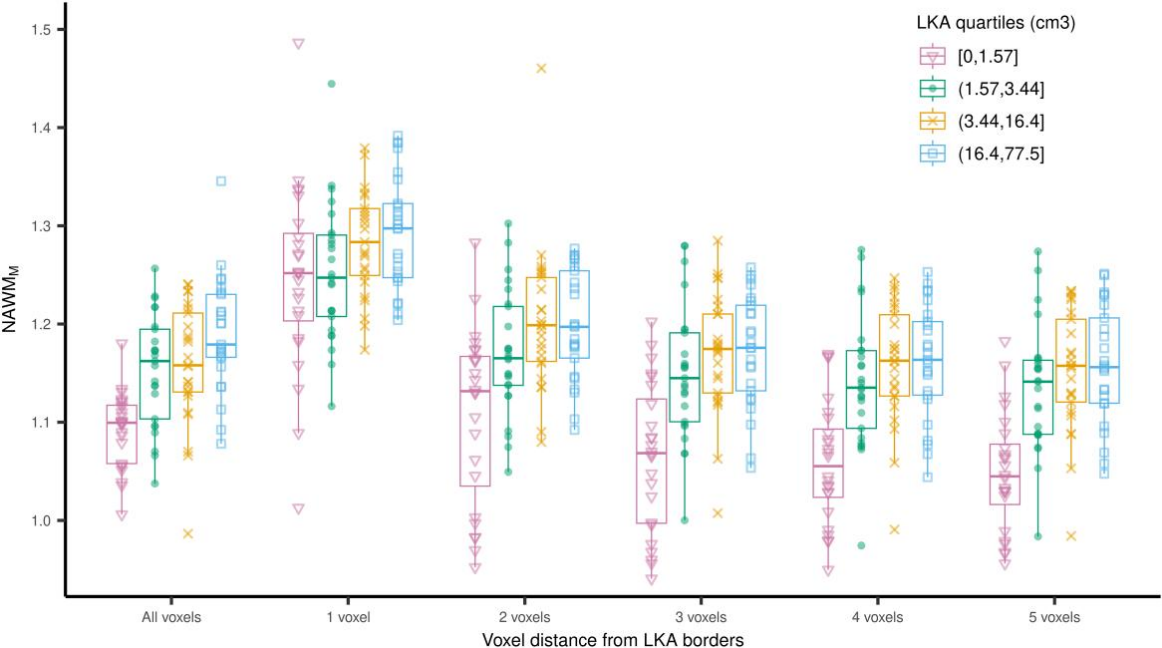
**NAWM_M_ at each leukoaraiosis (LKA) volume quartile (cm^3^), according to voxel distance from LKA borders.** The box plot shows the relationship between NAWM_M_ (*y*-axis) and LKA volume quartile (colour coding) in full NAWM and at each NAWM stratum according to voxel distance from LKA borders (*x*-axis). In a regression analysis using linear mixed modelling, the fixed effects analysis demonstrated an association between the LKA volume quartile and NAWM_M_ in the full NAWM region (*B* = 0.025; 95% confidence interval 0.019–0.030). When introducing random slopes to the model, the regression estimate for the full NAWM region did not change (*B* = 0.025; 95% confidence interval 0.016–0.033), and estimates for each NAWM stratum according to voxel distance from LKA were positive and numerically similar (from first to fifth voxel dilation: 0.014, 0.024, 0.027, 0.028, and 0.029). There was no difference between the models with and without random slopes in the likelihood test ratio (*P* = 0.14).

Voxel maps of leukoaraiosis frequency and NAWM_M_ are represented in the Montreal Neurological Institute space in Fig. 3. Patients with higher LKA_V_ had higher values of NAWM_M_, more conspicuously in the *centrum semiovale*. This increase in NAWM_M_ did not appear to correspond to the frequency maps of leukoaraiosis and was also present in voxels not outlining leukoaraiosis regions (Fig. 4).

## Discussion

In this study, we found that NAWM presents diffuse subtle white matter intensity changes that are related to the burden of leukoaraiosis. These findings are consonant with the increasing evidence that white matter damage related to small-vessel disease is not restricted to —and likely precedes— visible leukoaraiosis. We believe that our results suggest that white matter damage related to small-vessel disease is likely associated with extravascular leakage and free-water accumulation. On the other hand, the topographical distribution of FLAIR signal intensity changes was not exclusively adjacent to the regions of leukoaraiosis, thus suggesting that it possibly reflects a diffuse process and is not only a milder, incipient form of leukoaraiosis.

Our results are in agreement with studies that demonstrate diffuse pre-visual changes in white matter in patients with small-vessel disease. Both pathology and DTI studies have shown that the typical signature of leukoaraiosis is also found in NAWM.^12,14,15,25^ In pathology, this signature is characterized by demyelination, loosening of white matter fibres and accumulation of extracellular fluid.^26,27^ It has been proposed that the underlying aetiology of leukoaraiosis stems from chronic endothelial damage leading to blood–brain barrier disruption and eventually to extravascular leakage and indiscriminate infiltration of brain tissue with blood constituents.^28,29^ From a clinical perspective, a growing body of evidence has shown that DTI-derived markers of free water are related to the progression of leukoaraiosis and cognitive performance.^30-32^ Also, it has been shown that markers of blood–brain permeability are related to the long-term clinical outcome of patients with ischaemic stroke.^33,34^ We believe that such blood–brain barrier leakage could possibly account for the subtle FLAIR signal intensity changes observed in our study, given that FLAIR signal hyperintensity presumably reflects tissue free-water accumulation.

The topographical distribution of early white matter changes has been controversial in the literature. The first study to address this issue described DTI changes to be in close proximity to large areas of leukoaraiosis, thus leading to the coining of the term ‘white matter hyperintensity penumbra’.^35^ DTI changes and baseline FLAIR intensity within a perimeter of 8-mm distant from leukoaraiosis were predictors of incident leukoaraiosis in another study.^36^ On the other hand, Maniega *et al.*^25^ have demonstrated an association between leukoaraiosis severity and DTI changes in NAWM that was independent of proximity to leukoaraiosis. Also, studies evaluating markers of blood–brain barrier disruption have digressed from this proximity, penumbral pattern. In a longitudinal study with dynamic contrast-enhanced MRI in patients with Biswanger disease, blood–brain barrier disruption was related to baseline and incident leukoaraiosis, but the vast majority of voxels showing gadolinium enhancement were present not in leukoaraiosis regions, but within NAWM, and not in close proximity of either baseline or newly formed leukoaraiosis regions.^37^ Therefore, it appears that leukoaraiosis-related changes might develop diffusely across brain white matter and not exclusively surrounding established leukoaraiosis. Our results are in agreement with that hypothesis. In our regression analysis, the relationship between NAWM signal intensity and leukoaraiosis volume was similar between NAWM strata, with similar estimates for all NAWM strata except for the NAWM adjacent to leukoaraiosis, which showed a lower estimate. We hypothesize that in voxels adjacent to leukoaraiosis, NAWM signal intensity might have suffered from contamination from the adjacent diseased white matter, irrespective of the leukoaraiosis volume. Otherwise, our results indicate that NAWM signal increase related to leukoaraiosis appears to be diffuse. We believe that these subtle signal intensity changes on FLAIR, as well as DTI and blood–brain barrier changes, might not represent a milder stage of leukoaraiosis but rather reflect widespread aetiopathogenic processes underlying small-vessel disease.

Our study has some limitations. Our data are derived from MRI acquired for standard clinical care, with suboptimal resolution, anisotropic voxels and absence of DTI, perfusion imaging or gadolinium-enhanced sequences. Thus, the association of our findings with other markers of white matter damage could not be assessed. Also, we did not explore the potential relationship of NAWM signal intensity with other established markers of small-vessel disease, such as dilated perivascular spaces, cerebral microbleeds and superficial siderosis. We also need to mention that, as per a recent consensus statement on neuroimaging of small-vessel disease, there is a lack of reproducibility and comparability of the many available methods of white matter assessment, and the methods and results described herein should be reproduced in other settings to validate our conclusions. In addition, to minimize tissue misclassification in this suboptimal imaging setting, we selected a very conservative white matter mask for segmentation, which excluded juxtacortical white matter, a significant portion of subcortical white matter and white matter from the hemisphere contralateral to any acute or chronic infarct. This limits the validity of our findings as a true representation of diffuse white matter damage, and reproducing these results with more optimal imaging protocols and whole-brain white matter analysis is warranted. Moreover, it is possible that partial volume averaging might have contaminated NAWM signal intensity in voxels superiorly or inferiorly adjacent to leukoaraiosis voxels. In order to minimize this possibility, we performed 2D dilations. However, the possibility of vertically adjacent voxels of leukoaraiosis and NAWM cannot be excluded. While we cannot exclude this possibility, we would argue that if this was a major confounder of our results, then the relationship between NAWM signal intensity and leukoaraiosis volume would not have been found in NAWM strata distant from leukoaraiosis borders. Finally, as a retrospectively collected sample, we could not study the relationship of our findings with the evolution of white matter damage.

## Conclusion

The burden of leukoaraiosis is associated with subtle, diffuse FLAIR signal intensity changes in NAWM. Our data suggest that tissue fluid accumulation is possibly a harbinger of white matter damage related to small-vessel disease. Further studies are necessary to assess neuroimaging and pathology correlates and the clinical implications of these changes.

## Data Availability

The data that support the findings of this study are available on request from the corresponding author. The data are not publicly available due to the main research institution’s policy on confidentiality and patient data sharing. The data used for this study are available on request.
